# Editorial: New insights on botulism, botulinum neurotoxins, and botulinum toxin-producing clostridia

**DOI:** 10.3389/fmicb.2022.979653

**Published:** 2022-07-18

**Authors:** Fabrizio Anniballi, Theresa J. Smith, John W. Austin

**Affiliations:** ^1^Microbiological Food Safety and Food-Borne Diseases Unit, Department of Food Safety, Nutrition and Veterinary Public Health, National Reference Centre for Botulism, Istituto Superiore di Sanità, Rome, Italy; ^2^Pathogen and Microbiome Institute, Northern Arizona University, Flagstaff, AZ, United States; ^3^Botulism Reference Service for Canada, Microbiology Research Division, Bureau of Microbial Hazards, Food Directorate, Health Products and Food Branch, Ottawa, ON, Canada

**Keywords:** botulinum neurotoxins, botulinum toxin-producing clostridia, wound botulinum, animal botulism, CRISPR-Cas systems

Botulism is a life-threatening disease affecting humans, and many warm-blooded animals and fishes. Botulism is caused by botulinum neurotoxins (BoNTs) produced by anaerobic, spore-forming bacteria belonging to the genus *Clostridium*. Although the disease in humans is rare, each botulism case constitutes a public health emergency. A prompt clinical diagnosis and laboratory confirmation are essential for the correct management of patients and activating proper public health measures addressed to avoid the further cases. Conversely, botulism is more common in animals and may result in a high mortality rate, eliciting environmental, and economic concerns.

Both human and animal botulism have been classified into different forms based on route of exposure to BoNTs. Foodborne and iatrogenic botulism are based on ingestion/injection of toxin, while infant botulism and wound botulism are toxicoinfections that result after the growth and expression of bacteria within the body. On a worldwide basis, food-borne botulism, due to the consumption of foods contaminated with preformed toxins, is the most widely recognized form. Home-prepared foods are mainly responsible for sporadic cases, and small outbreaks occur primarily at the familiar level. Commercially produced foods may cause large outbreaks affecting multiple regions or countries. Since botulism can result in respiratory failure requiring hospitalization in intensive care units for weeks or months, these latter outbreaks pose a challenge for local and regional health systems. As highlighted by Lúquez et al. in their manuscript on food-borne botulism outbreaks that occurred in the US from 2001 to 2017, modern hospitals can admit to intensive care units only a limited number of patients. Thus, a local outbreak of foodborne botulism could easily overwhelm the hospital system with worried asymptomatic or paucisymptomatic people, delaying ventilatory assistance and botulinum antitoxin administration to those who need it most. Local health systems should consider this when implementing and revising their emergency preparedness plans. For optimal management of a botulism case/outbreak, it is fundamental that early clinical diagnosis and rapid activation of the epidemiological surveillance system is aimed at identifying the implicated food vehicle and avoiding new cases.

Clinical diagnosis might be difficult because of the rarity of botulism and because, at the onset, symptoms and clinical signs may be non-specific. The difficulty increases if the patient is a drug user affected by wound botulism because some neurologic signs may be confused with the drug effects. In wound botulism cases, the physician's awareness and timely diagnosis are crucial to adopting suitable treatments, such as botulinum antitoxin administration and debridement of the wound. In their manuscript, Middaugh et al. confirmed that early clinical diagnosis and prompt administration of antitoxin prevents respiratory failure and reduces hospital stay. In addition, they highlighted the importance of wound botulism surveillance among close contacts with persons who injected drugs, as well as the importance of health alerts to raise clinicians' awareness of wound botulism. Molecular surveillance of microbial isolates could help identify the source of contamination in injectable drugs. Since *Clostridium botulinum* is an environmental microorganism, spores may contaminate the drug at different stages of production and processing, such as where the raw materials were grown and during cutting and diluting. Contamination at the site of use also cannot be ruled out. To trace the contamination source of drug consumed by an injection drug user affected by wound botulism, Halpin et al. studied the phylogenetic relatedness of *C. botulinum* isolated in Hawaii from wound and infant botulism cases. *C. botulinum* strains isolated from infant botulism cases were chosen because they have been considered representative of the environment in which cases occurred. As the main finding, Halpin et al. found a high homology among isolates, supporting the hypothesis that the organism implicated in wound botulism in this case contaminates the drug paraphernalia or the wound itself locally rather than at the production site or during transport.

Molecular sub-typing of isolated strains is crucial for source attribution. In this respect, several molecular biology techniques have been developed. The Multiple Locus Variable-number of tandem repeat Analysis (MLVA) was successfully adopted by Souillard et al. during their investigations of an outbreak of cattle botulism. Souillard et al. identified a poultry house as the source of contamination in a massive type D/C cattle botulism outbreak on a mixed dairy and broiler farm in France. This was accomplished by monitoring carriage in the broilers, the ventilation system of the poultry house, and contamination of equipment from the hatchery used for delivering the chicks. As a second-generation sub-typing technique, MLVA is less discriminating than Whole Genome Sequencing (WGS); however, as a PCR-based technique, it can be performed using only a tiny amount of DNA template. If a species-specific scheme is used (as Souillard et al. have done), MLVA avoids the strain isolation and purification step that is time-consuming and particularly challenging for the microbial agents of animal botulism.

For genomic characterization of strains isolated from a cluster of infant botulism type A cases, Gladney et al. used WGS single-nucleotide polymorphism (SNP) analysis. Using Lyve-SET high-quality SNP analysis, they were able to differentiate strains isolated in their cluster from other *C. botulinum* type A(B), demonstrating the high-resolution level of this technique. Another molecular approach adopted for bacterial sub-typing uses Clustered Regularly Interspaced Short Palindromic Repeats (CRISPRs) analysis. CRISPR and CRISPR-associated proteins (Cas) are a prokaryotic adaptative immunity and regulatory system that interferes with invading phages and plasmids and can be used to gain direct insight into horizontal gene transfer events. The analysis of CRISPR-Cas systems was carried out by Le Gradiet et al. testing 58 *Clostridium novyi sensu lato* genospecies (responsible for animal botulism), while Wentz et al. profiled endogenous CRISPR-Cas systems from 241 Group I *C. botulinum* (proteolytic strains) and *Clostridium sporogenes* genomes. Le Gradiet et al. conducted their studies to explore CRISPR-Cas systems in the *Clostridium novyi sensu lato* strains to evaluate their presence, determine their characteristics, and explore the protospacer origins to gain insight into the mobile genetic elements (MGE) interacting with this taxon. They found that CRISPR-Cas systems are numerous in *Clostridium novyi sensu lato* strains and may present in the bacterial chromosome or MGE. The components carrying out CRISPR-Cas systems seem to have been recruited as anti-MGE systems and for inter-MGE conflicts to protect mainly against a restricted number of MGEs. In their investigations, Wentz et al. found that the *bont* gene cluster was not directly targeted by endogenous CRISPR-Cas systems in Group I *C. botulinum* and *C. sporogenes*. An extensive study on the evolution of *bont* gene clusters harbored by Group I *C. botulinum* was performed by Smith et al. They found that strains isolated in the northern hemisphere harbor *bont* gene clusters containing *ha* genes, whilst those isolated in the southern hemisphere primarily harbor *bont* gene clusters containing *orfX* genes. In addition, they found that the movement of *bont* gene clusters into non-neurotoxigenic clostridia is a one-way process that occurs *via* introduction within extrachromosomal plasmids followed by chromosomal integration.

Among CRISPR-Cas systems, CRISPR-Cas9 is widely used as a versatile tool to perform gene editing. In this respect, Mertaoja et al. published an elegant paper which exemplified CRISPR-Cas9 bookmark technology to construct mutants suitable to investigate the sporulation mechanism in Group II *C. botulinum* (non-proteolytic) strains. Moreover, they demonstrated the potential of the bookmark technology in functional studies in which a chromosomal gene is replaced, in a two-step process, with a derivatized copy.

In summary, the articles included in this Research Topic provide an overview of current research activities in the botulism field and illustrate the growing use of genomic techniques in epidemiologic investigations as well as basic research applications. SNP analysis of whole-genome sequences was used to investigate the relatedness of wound botulism isolates in Hawaii and infant botulism isolates in Colorado, and MLVA studies were used to trace the source of contamination in an outbreak involving cattle. Genomic analysis of a range of clostridial strains having *orfX*+ *bont* gene clusters provided insights as to the evolutionary origins and spread of these strains. The realization of the importance of CRISPR-Cas systems in manipulating mobile genetic elements within bacteria has inspired articles investigating the links between these systems and mobile genetic elements, including exploitation of these systems to better understand the factors contributing to spore formation in botulinum neurotoxin-producing clostridia. These articles highlight the main challenges and propose solutions to improve management and control of the disease, stressing the power of the newer techniques as suitable tools to enhance botulism prevention measures and address future trends in botulism research.

## Author contributions

All authors listed have made a substantial, direct, and intellectual contribution to the work and approved it for publication.

## Conflict of interest

The authors declare that the research was conducted in the absence of any commercial or financial relationships that could be construed as a potential conflict of interest.

## Publisher's note

All claims expressed in this article are solely those of the authors and do not necessarily represent those of their affiliated organizations, or those of the publisher, the editors and the reviewers. Any product that may be evaluated in this article, or claim that may be made by its manufacturer, is not guaranteed or endorsed by the publisher.

